# The impact of a heart failure management protocol based on a hospital-community pharmacist collaboration

**DOI:** 10.1186/s40780-025-00426-5

**Published:** 2025-03-12

**Authors:** Junichi Terashima, Takahiro Kambara, Eisei Hori, Masahiro Fukatsu, Yukina Ichiki, Eri Oki, Risako Koketsu, Rika Taguchi, Suzuka Mii, Ryoka Hiro, Teruhiro Sakaguchi, Hiroyuki Osanai, Tomoya Tachi, Tadashi Suzuki

**Affiliations:** 1 Department of Pharmacy, Tosei General Hospital, 160, Nishioiwake-cho, Seto, Aichi 489- 8642 Japan; 2 Department of Clinical Pharmacy, Graduate School of Pharmaceutical Sciences, Nagoya City University, 3-1, Tanabedori, Mizuho-ku, Nagoya, Aichi 467-8603 Japan; 3 Department of Cardiovascular Medicine, Tosei General Hospital, 160, Nishioiwake-cho, Seto, Aichi 489-8642 Japan; 4 Education and Research Center for Clinical Pharmacy, Faculty of Pharmaceutical Sciences, Nagoya City University, 3-1, Tanabedori, Mizuho-ku, Nagoya, Aichi 467-8603 Japan

**Keywords:** Heart failure, Management protocol, Community pharmacist, Collaboration

## Abstract

**Background:**

Heart failure has a high readmission rate, but interventions by multiple professionals are effective. Although there is growing interest in the management of heart failure by community pharmacists in Japan, no effective method has been developed. We created and verified the effectiveness of a novel heart failure management protocol that community pharmacists could utilize.

**Methods:**

This study included 68 patients (80.8 ± 11.8 years; male, 60.3%) diagnosed with heart failure who was admitted to our hospital between March 2022 and September 2023. A protocol was developed for the regular follow-up of patients and responses to exacerbations, in collaboration with pharmacists. Patients who were able to receive follow-up from community pharmacists were included in the intervention group, otherwise they were included in the control group. The primary endpoint was readmission, and the secondary endpoint was a composite of readmission, all-cause death, hospitalization, and dialysis due to dehydration or renal dysfunction.

**Results:**

The Kaplan–Meier analysis (*p* = 0.021) and Cox model (hazard ratio: 0.28, 95% confidence interval: 0.09–0.89, *p* = 0.031) revealed a significantly lower incidence of heart failure readmission within 360 days in the intervention group than in the control group.

**Conclusions:**

The implementation of a heart failure management protocol that provides clear guidance on appropriate patient management enabled pharmacists to effectively reduce the likelihood of adverse events, such as heart failure readmission.

**The registration number:**

UMIN000046750, registered on February 1, 2022.

## Introduction

Heart failure has an extremely high readmission rate with one of the global challenges being prolonging of home care [1–4]. A multidisciplinary approach from multiple perspectives is important because the factors that lead to readmission are extremely diverse. Previous reports have suggested that continuous intervention by a multidisciplinary team may lead to a reduction in heart failure readmission rates [1–4]. Inappropriate medication management is believed to be a significant factor to readmissions [5]; however, substantial evidence from overseas studies indicates that community interventions by pharmacists effectively enhance medication adherence [6–12].

Continuous follow-up of patients with heart failure has been garnering attention in Japan; hence, from 2024, the follow-up of patients with heart failure by community pharmacists has become a new target in the medical field. Pharmacist intervention using pharmacological knowledge is an effective approach in patient management; however, there is no clear method for pharmacists to manage heart failure in Japan. Furthermore, there is lacking evidence regarding the effectiveness of this approach in reducing the incidence of heart failure readmissions. In collaboration with physicians, a heart failure management protocol was developed for community pharmacists to monitor patients and provide appropriate pharmaceutical interventions, which includes additional management procedures for the use of diuretics by community pharmacists. Loop diuretics are often used to treat organ congestion symptoms, such as shortness of breath and edema, in patients with heart failure. Although loop diuretics can help heart failure patients [13], these can also cause dehydration and renal dysfunction, and impair long-term prognosis [14, 15]; therefore, these should be used with caution. Temporary use of diuretics may help manage the symptoms of weight gain with good patient education [16]; however, relying solely on patients for diuretic management may lead to certain risks.

The objective of this prospective interventional study was to develop a community-based heart failure management protocol in collaboration with pharmacists, and to evaluate the effects of this protocol on outcomes, such as heart failure readmission, all-cause mortality, hospitalization due to renal dysfunction or dehydration, and dialysis initiation. The protocol specified that hospital pharmacists were responsible for facilitating collaboration between attending physicians and pharmacists. In addition, community pharmacists were tasked with providing continuous follow-up regarding heart failure symptoms and adherence, and they were permitted to make decisions regarding the temporary use of diuretics in accordance with the established protocol.

## Methods

### Study design

This was an open-label, single-center, between-group, prospective interventional study of patients admitted for heart failure treatment at the Tosei General Hospital (Japan) between March 2022 and September 2023. The study conformed to the principles outlined in the Declaration of Helsinki and was approved by the Tosei General Hospital Medical Ethics Committee (approval number: 1021). Written informed consent was obtained from all participants. This study was registered at UMIN-CTR (UMIN study ID: UMIN000046750).

### Studied patients

The study population consisted of patients who were at least 18 years old at the time of consent and had been diagnosed with stage C heart failure according to the American College of Cardiology Foundation/American Heart Association.

It was a prerequisite for patients to visit the cardiology department of the Tosei General Hospital as an outpatient following discharge from the hospital to participate in this study. Furthermore, it was important that each patient consistently received medications from the same pharmacy.

The exclusion criteria included patients who were deemed unsuitable for the study by the attending physicians owing to the presence of significant comorbidities, including but not limited to complications arising from disorders affecting the kidney, liver, blood, respiratory, digestive, neuropsychiatric, or metabolic systems. Patients who demonstrated difficulty in visiting Tosei General Hospital or those who explicitly declined to participate were excluded. Finally, patients who were unable to provide informed consent were also excluded.

Patients who were able to receive follow-up from community pharmacists were included in the intervention group. Patients received interventions in accordance with the protocol established in this study. The remaining patients were assigned to the control group and received general treatment.

### Data collection and follow-up

Patient backgrounds, including sex, age, body mass index (BMI), laboratory values, and medications at discharge, were collected. Medical history regarding diabetes and heart failure-related events, such as hospitalization for heart failure, myocardial infarction, atrial fibrillation, coronary artery bypass grafting (CABG), and percutaneous coronary intervention (PCI), were obtained from the medical records of patients.

Detailed data on the observation period of patients were collected after discharge from the hospital. In addition to the electronic medical records and laboratory results, pharmacy reports, communications, and records were collected and followed-up for 360 days for the occurrence of various events.

### Intervention

**Fig. 1:**
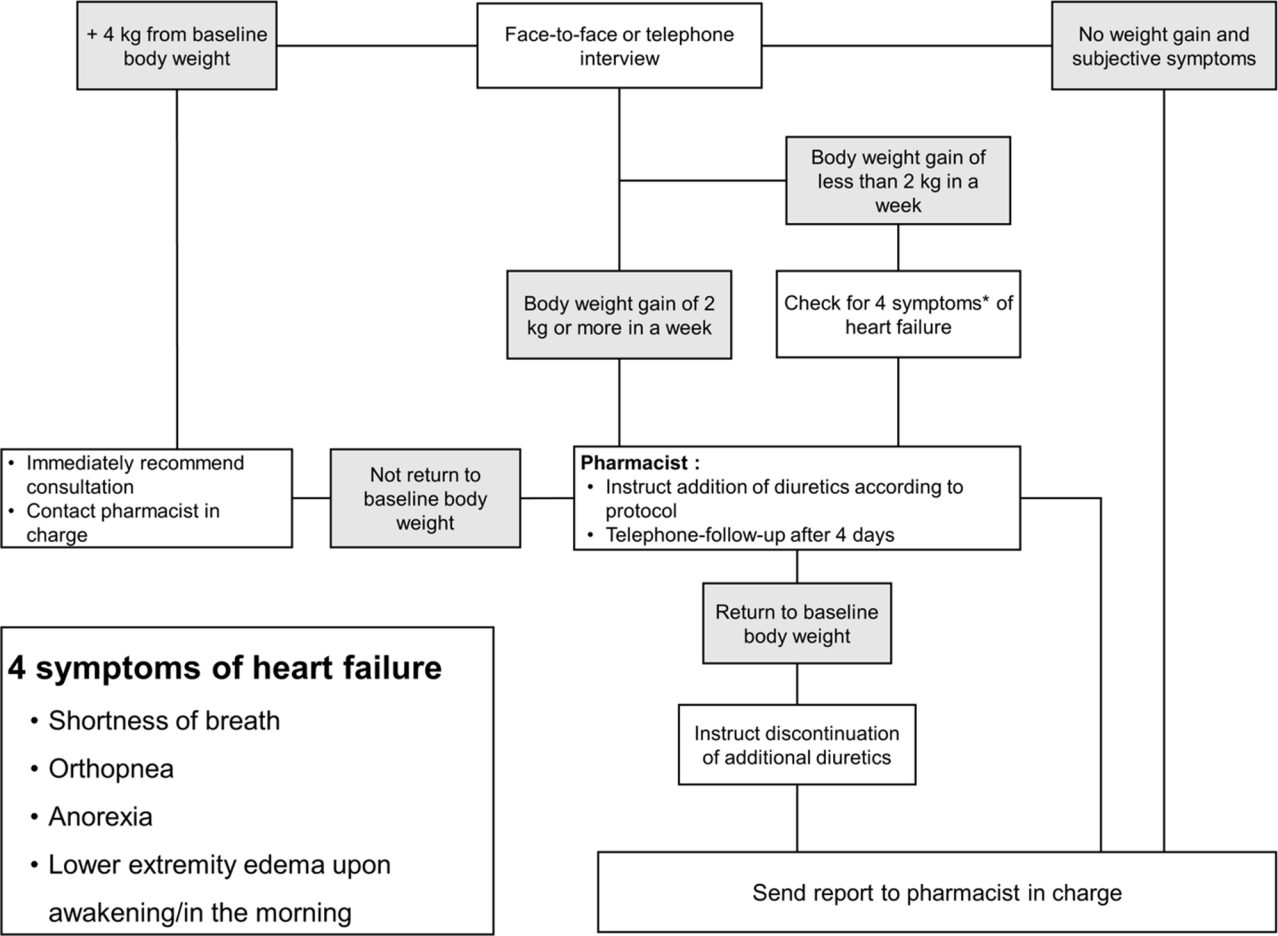
Algorithm for the heart failure management protocol

The intervention group received follow-up guidance from community pharmacists during the outpatient period. The protocol for heart failure management is illustrated in Fig. 1. The protocol defines the requirements for community pharmacists to provide support, and it also specifies the actions to observe and reporting procedures when signs of worsening heart failure are present.

Before the follow-up period, community pharmacists received basic training regarding heart failure and the protocol. It was recommended that follow-ups be conducted on a regular basis, with each interval lasting for a minimum of 2 weeks. Patient follow-ups were conducted face-to-face or through telephone interviews. In addition to verifying medication adherence and monitoring the weight and blood pressure of patients, community pharmacists also evaluated the presence or absence of heart failure symptoms, including dyspnea, orthopnea, appetite, and lower-limb edema, using a questionnaire. Furthermore, community pharmacists ascertained whether there has been an exacerbation of heart failure based on deviations from the pre-established target weight and the presence of heart failure symptoms.

At the time of protocol initiation and at the time of obtaining consent, the physician set a target weight for heart failure management at the time of discharge. This protocol determined the worsening of heart failure by observing the symptoms and weight of patients for over a week. If the patient weight increased by 2 kg, or by 1 kg with the presence of heart failure symptoms, or by less than 1 kg but the community pharmacist deemed it to be associated with the heart, the community pharmacist administered 4 mg of torasemide. Community pharmacists then recommended that patients take additional doses of torasemide for up to 3 days to avoid having to take torasemide for an extended period. Following the 3 days, the community pharmacists contacted the patients to ascertain the status of the medication and efficacy of the treatment. The community pharmacists notified Tosei General Hospital and encouraged patients to seek immediate medical care if patients gained ≥ 4 kg above the pre-established target weight.

In addition to routine follow-ups, community pharmacists provided guidance to patients and their families regarding self-monitoring of body weight and heart failure symptoms. Patients were advised to consult a community pharmacist in the event of weight gain of ≥ 2 kg within a week, ≥ 4 kg above the target weight, or the onset of any of the four symptoms of heart failure. Patient responses were gathered during follow-up procedures, and consultations were conducted when necessary.

It was mandated that community pharmacists submit a tracing report (TR) to hospital pharmacists whenever they conducted regular or emergency responses, or prior to and following the administration of supplementary torasemide. If they anticipated difficulty responding to or receiving urgent information, they were obligated to contact hospital pharmacists via telephone.

To guarantee the inclusion of all obligatory data, specific data fields were established for reporting purposes. Hospital pharmacists who received the reports evaluated the content and implemented the necessary adjustments, which included providing feedback to doctors and coordinating treatment plans between local pharmacists and doctors.

The TRs were transmitted to the Tosei General Hospital via facsimile. Hospital pharmacists reviewed the TRs and took appropriate actions, such as storing them in the pharmaceutical department, contacting the physicians, entering the information into the medical record, and providing guidance to the community pharmacists. In cases of immediate need, pharmacists contacted physicians and informed the community pharmacists about their responses.

In response to reports from community pharmacists, hospital pharmacists engaged in consultations with physicians regarding the implementation of measures such as intensified observation or resetting of the target weight, as deemed appropriate by the physicians. Hospital pharmacists then disseminated the agreed-upon measures to community pharmacists. In cases where the hospital pharmacist determined that immediate action was necessary, they informed the doctor, who would then decide on the appropriate course of action. Consequently, if an emergency consultation was deemed essential, the hospital pharmacist contacted the patient via the pharmacy and coordinated with each department to arrange an outpatient consultation.

Community pharmacists collaborated with the Tosei General Hospital to administer treatment, whereas hospital pharmacists evaluated the adherence of community pharmacists to the protocol and ensured effective internal communication and coordination in cases of urgent consultations.

In contrast, the control group was not subjected to any restrictions on the usual patient follow-up or transmission of TR data to the hospital.

### Definition of primary and secondary outcomes

The primary endpoint was the first readmission for worsening chronic heart failure, and the secondary endpoint was a composite of the first readmission for worsening chronic heart failure, all-cause death, hospitalization, or dialysis due to dehydration or renal dysfunction.

### Statistical analysis

Continuous variables were expressed as mean ± standard deviation or median, 25/75th percentile (interquartile range). Categorical variables were expressed as frequencies and percentiles. Baseline variables were compared between the two groups using Student’s t-test or Mann–Whitney U test for continuous variables, and Fisher’s exact test for categorical variables.

The follow-up rate of pharmacists in the intervention group was calculated as the ratio of the actual to the theoretical number of days of follow-up during the observation period, with ± 3 days as the allowable range from one follow-up day every 14 days.

**Fig. 2:**
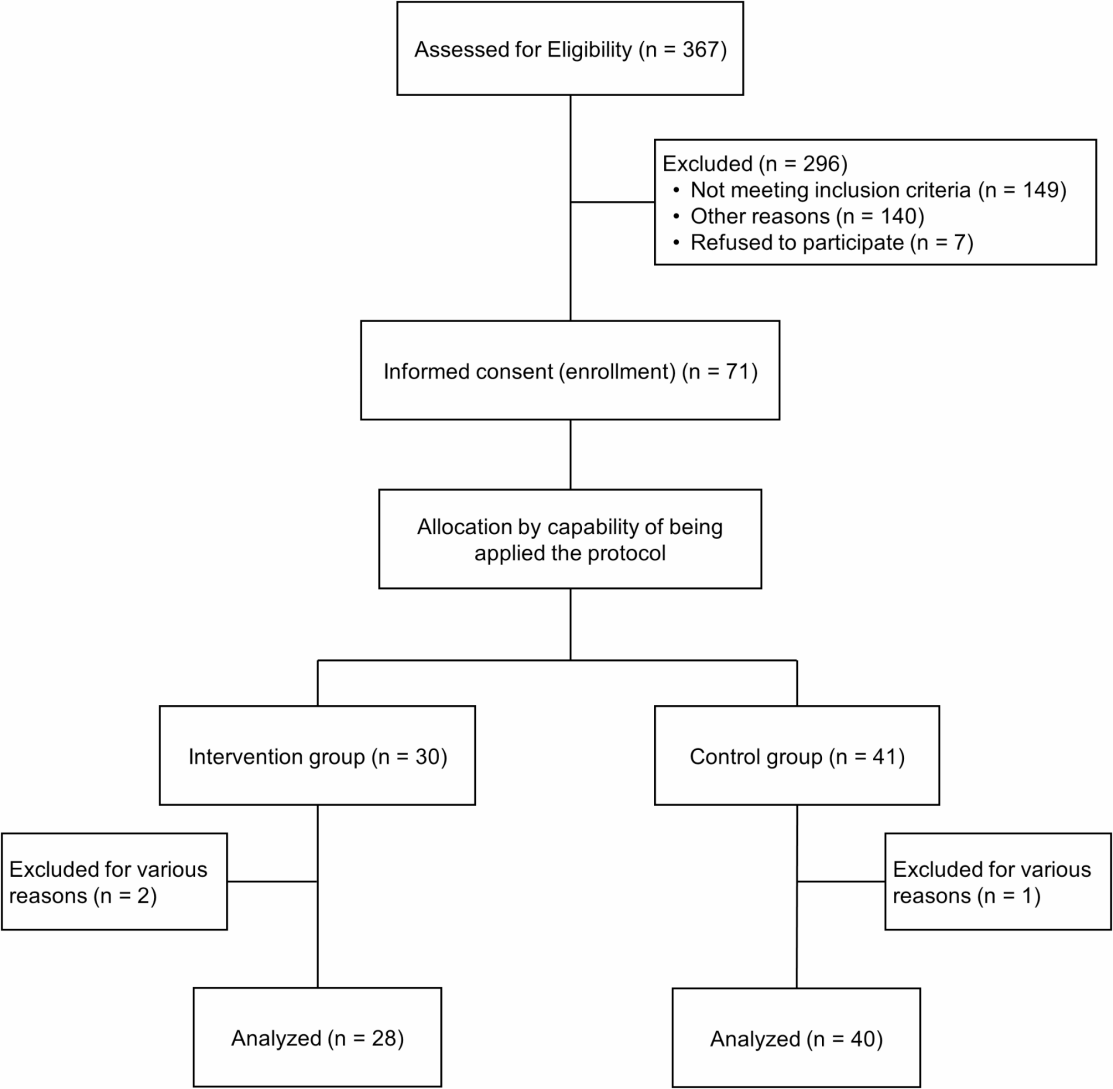
Flowchart of the study. PPS, per protocol set

First readmission events after discharge, all-cause mortality, and composite endpoints were compared between the two groups in relation to the heart failure management protocol developed in this study using Kaplan–Meier curves and log-rank tests, and the corresponding hazard ratio (HR) was estimated using the COX proportional hazard model, with 95% confidence intervals (CI). To accurately validate the effectiveness of this protocol, the intervention group was terminated in case of any deviation from the selection criteria or discontinuation of hospital visits, and in case of any change in instructions from this protocol or loss to follow-up for various reasons during the follow-up period. Per protocol set (PPS) analysis was performed when patients in the intervention group were terminated (Fig. 2).

For the PPS, 12 baseline covariates (age, gender, history of heart failure hospitalization, ischemic heart disease, atrial fibrillation, diabetes mellitus, BMI [kg/m^2^] at discharge, serum albumin level [g/dL], estimated glomerular filtration rate [mL/min/1.73 m^2^], hemoglobin level [g/dL]) were considered factors that affected the current intervention, heart failure readmission, N-terminal pro-B-type natriuretic peptide level (NT-proBNP; pg/mL), and left ventricular ejection fraction (LVEF; %). Propensity scores (PS) were calculated using logistic regression analysis of baseline variables, and 23 pairs from both groups were matched and analyzed by 1:1 nearest neighbor matching with 0.20 times the standard deviation of PS as the caliper. Statistical significance was set at a two-sided *p* < 0.05. All statistical analyses were performed using SPSS version 25 (Chicago, IL, USA).

## Results

### Analyzed patients

A total of 367 patients who were admitted to the public Tosei General Hospital for heart failure treatment between March 2022 and September 2023 were screened, and written consent was obtained from 71 patients. Of the total, 30 and 41 patients were included in the intervention and control groups, respectively; however, two patients in the intervention group were excluded because the intervention was not properly initiated by the pharmacist, and one patient in the control group was excluded from the evaluation because of unsuitable selection criteria. Finally, 28 and 40 patients in the intervention and control groups were analyzed (Fig. 2).

### Baseline characteristics

Baseline patient background was documented at the time of discharge when consent was obtained. The mean age of the 68 patients was 80.8 ± 11.8 years, with 60.3% of the cohort being male. The baseline characteristics were comparable between the two groups, which included the unadjusted BMI, history of heart failure hospitalization, myocardial infarction, atrial fibrillation, CABG, PCI, diabetes mellitus, serum albumin level, renal function (blood urea nitrogen, creatinine, estHimated glomerular filtration rate, and creatinine clearance), NT-proBNP, LVEF, and other laboratory values (albumin and hemoglobin). The PS was calculated using logistic regression analysis of the baseline patient characteristics that are presented in Table 1. The patient characteristics in the adjusted dataset obtained after 1:1 nearest-neighbor matching are shown in Table 2.

**Table 1:** Baseline patient demographics

	Overall cohort (*n* = 68)	Intervention group (*n* = 28)	Control group (*n* = 40)	*p*
Age (years, mean ± SD)	80.8 ± 11.8	80.0 ± 8.9	81.3 ± 10.8	0.662
Male, n (%)	41 (60.3)	15 (53.6)	26 (65.0)	0.451
Living alone, n (%)	8 (11.8)	6 (21.4)	2 (5.0)	0.057
Medical history, n (%)				
Diabetes mellitus	20 (29.4)	8 (28.6)	12 (30.0)	1.000
Previous HF admission	27 (39.7)	13 (46.4)	14 (35.0)	0.451
Ischemic heart disease	29 (42.6)	13 (46.4)	16 (40.0)	0.627
Artral fibrillation	50 (73.5)	19 (67.9)	31 (77.5)	0.375
Previous CABG	4 (5.9)	3 (10.7)	1 (2.5)	0.298
Previous PCI	21 (30.9)	10 (35.7)	11 (27.5)	0.595
Physical examination				
Systolic blood pressure (mmHg, mean ± SD)	111.6 ± 16.3	113.4 ± 17.4	110.3 ± 15.6	0.447
Diastolic blood pressure (mmHg, mean ± SD)	65.2 ± 11.5	65.8 ± 11.0	64.8 ± 11.9	0.600
Body mass index (kg/m^2^), n (%)	20.7 ± 3.9	21.0 ± 3.1	20.5 ± 4.4	0.252
LVEF	50.5 ± 15.1	49.6 ± 13.6	51.2 ± 5.3	0.570
LVEF < 50, n (%)	31 (45.6)	14 (50.0)	17 (42.5)	0.624
Albumin (g/dL, mean ± SD)	3.2 ± 0.4	3.2 ± 0.4	3.2 ± 0.5	0.549
Creatinine (mg/dL, mean ± SD )	1.29 ± 0.6	1.31 ± 0.6	1.27 ± 0.6	0.847
Blood urea nitrogen (mg/dL, mean ± SD)	29.2 ± 14.4	28.5 ± 12.4	29.7 ± 15.8	0.945
eGFR (mL/min/1.73m^2^, mean ± SD^2^)	45.3 ± 21.0	43.0 ± 18.7	47.0 ± 22.5	0.455
Hemoglobin (g/dL, mean ± SD)	12.1 ± 2.1	12.2 ± 2.0	12.0 ± 2.2	0.566
Creatinine clearance (mL/min, mean ± SD)	37.7 ± 22.9	36.9 ± 21.4	38.2 ± 24.2	0.921
NT-proBNP (pg/mL), median (IQR)	2380 (1335–4549)	3033 (1622–6306)	1749 (1226–3736)	0.100
Medication, n (%)				
Aspirin	11 (16.2)	3 (10.7)	8 (20.0)	0.505
OACs	40 (58.8)	16 (57.1)	24 (60.0)	1.000
Beta-blocker	53 (77.9)	21 (75.0)	32 (80.0)	0.768
ACEi/ARB/ARNI	44 (64.7)	20 (71.4)	24 (60)	0.441
MRA	38 (55.9)	19 (67.9)	19 (47.5)	0.137
SGLT2i	30 (44.1)	10 (35.7)	20 (50.0)	0.322
Loop diuretics	58 (85.3)	23 (82.1)	35 (87.5)	0.730

SD, standard deviation; HF, heart failure; CABG, coronary artery bypass grafting; PCI, percutaneous coronary intervention; IQR, interquartile range; LVEF, left ventricle ejection Fraction; eGFR, estimated glomerular filtration rate; NT-proBNP, N-terminal pro brain natriuretic peptide; OACs, oral anticoagulants; ACEi, angiotensin converting enzyme inhibitors; ARB, angiotensin receptor blockers; ARNI, angiotensin receptor neprilysn inhibitors; MRA, mineralocorticoid receptor antagonists; SGLT2i, sodium-glucose cotransport-2 inhibitor

**Table 2:** Patient demographics after propensity score matching (fully adjusted model)

	Overall cohort (*n* = 46)	Intervention group (*n* = 23)	Control group (*n* = 23)	*p*
Age (years, mean ± SD)	80.2 ± 13.1	80.4 ± 11.8	80.0 ± 14.7	0.965
Male, n (%)	22 (47.8)	11 (47.8)	11 (47.8)	1.000
Living alone, n (%)	6 (13.0)	5 (21.7)	1 (4.3)	0.187
Medical history, n (%)				
Diabetes mellitus	12 (26.1)	7 (30.4)	5 (21.7)	0.738
Previous HF admission	20 (43.5)	10 (43.5)	10 (43.5)	1.000
Ischemic heart disease	20 (43.5)	11 (47.8)	9 (39.1)	0.767
Artral fibrillation	34 (73.9)	16 (69.6)	18 (78.3)	0.738
Previous CABG	4 (8.7)	3 (13.0)	1 (4.3)	0.608
Previous PCI	14 (30.4)	8 (34.8)	6 (26.1)	0.749
Physical examination				
Systolic blood pressure (mmHg, mean ± SD)	113.3 ± 18.0	113.9 ± 18.5	112.6 ± 17.9	0.852
Diastolic blood pressure (mmHg, mean ± SD)	66.2 ± 12.4	65.6 ± 11.6	66.7 ± 13.3	0.869
Body mass index (kg/m^2^), n (%)	21.4 ± 4.1	21.0 ± 3.2	21.9 ± 4.9	0.852
LVEF	49.5 ± 14.8	49.7 ± 13.2	49.3 ± 16.6	0.921
LVEF < 50, n (%)	21 (45.7)	11 (47.8)	10 (43.5)	1.000
Albumin (g/dL, mean ± SD)	3.2 ± 0.4	3.2 ± 0.4	3.3 ± 0.4	0.300
Creatinine (mg/dL, mean ± SD )	1.36 ± 0.59	1.32 ± 0.60	1.40 ± 0.59	0.391
Blood urea nitrogen (mg/dL, mean ± SD)	31.0 ± 14.4	29.1 ± 12.7	32.8 ± 16.1	0.538
eGFR (mL/min/1.73m^2^, mean ± SD)	40.7 ± 17.9	42.7 ± 19.9	38.8 ± 15.8	0.645
Hemoglobin (g/dL, mean ± SD)	12.0 ± 2.0	11.7 ± 1.8	12.3 ± 2.3	0.495
Creatinine clearance (mL/min, mean ± SD)	35.7 ± 23.7	35.6 ± 20.5	35.9 ± 27.0	0.637
NT-proBNP (pg/mL), median (IQR)	2809 (1596–5131)	3165 (1704–6078)	2428 (1322–3907)	0.307
Medication, n (%)				
Aspirin	8 (17.4)	3 (13.0)	5 (21.7)	0.699
OACs	26 (56.5)	13 (56.5)	13 (56.5)	1.000
Beta-blocker	37 (80.4)	18 (78.3)	19 (82.6)	1.000
ACEi/ARB/ARNI	31 (67.4)	17 (73.9)	14 (60.9)	0.530
MRA	23 (50.0)	14 (60.9)	9 (39.1)	0.238
SGLT2i	18 (39.1)	6 (26.1)	12 (52.2)	0.130
Loop diuretics	38 (82.6)	18 (78.3)	20 (87.0)	0.699

We adjusted for 12 baseline covariates that were considered as factors that affected the current intervention, heart failure readmission, N-terminal pro-B-type natriuretic peptide level (pg/mL), and left ventricular ejection fraction (%)

SD, standard deviation; HF, heart failure; CABG, coronary artery bypass grafting; PCI, percutaneous coronary intervention; IQR, interquartile range; LVEF, left ventricle ejection Fraction; eGFR, estimated glomerular filtration rate; NT-proBNP, N-terminal pro brain natriuretic peptide; OACs, oral anticoagulants; ACEi, angiotensin converting enzyme inhibitors; ARB, angiotensin receptor blockers; ARNI, angiotensin receptor neprilysn inhibitors; MRA, mineralocorticoid receptor antagonists; SGLT2i, sodium-glucose cotransport-2 inhibitor

### Interventions

The follow-up periods The follow-up in the intervention group averaged 86.9%, with 84.6% of patients achieving at least 75% follow-up. One patient was difficult to follow-up because the patient did not fully understand the protocol, and the observation period ended when regular follow-up was deemed impossible. The main reason for no follow-up in other cases was the absence of the patient and pharmacist, or failure to respond to contact by the patient.

In the intervention group, the pharmacist provided information and consultation, in addition to regular post-follow-up reports and TRs, whereas in the control group, no TRs were conducted during the observation period. Twelve of 28 patients received one or more additional doses of diuretics at the discretion of the pharmacists, and there were no adverse events, such as renal dysfunction or dehydration, associated with these additional doses. In one case, the pharmacist contacted the patient to determine the efficacy of the medication, but the diuretic was not used as directed, which resulted in an unplanned visit and hospitalization. In another case, the diuretic was started as directed by the pharmacist, but the patient did not improve and was hospitalized.

In four cases, PPS was terminated because the physician provided diuretic instructions that differed from the protocol instructions. One of the patients who discontinued treatment was hospitalized for renal dysfunction 84 days after the change in diuretic instructions.

### Readmission due to heart failure

**Fig. 3:**
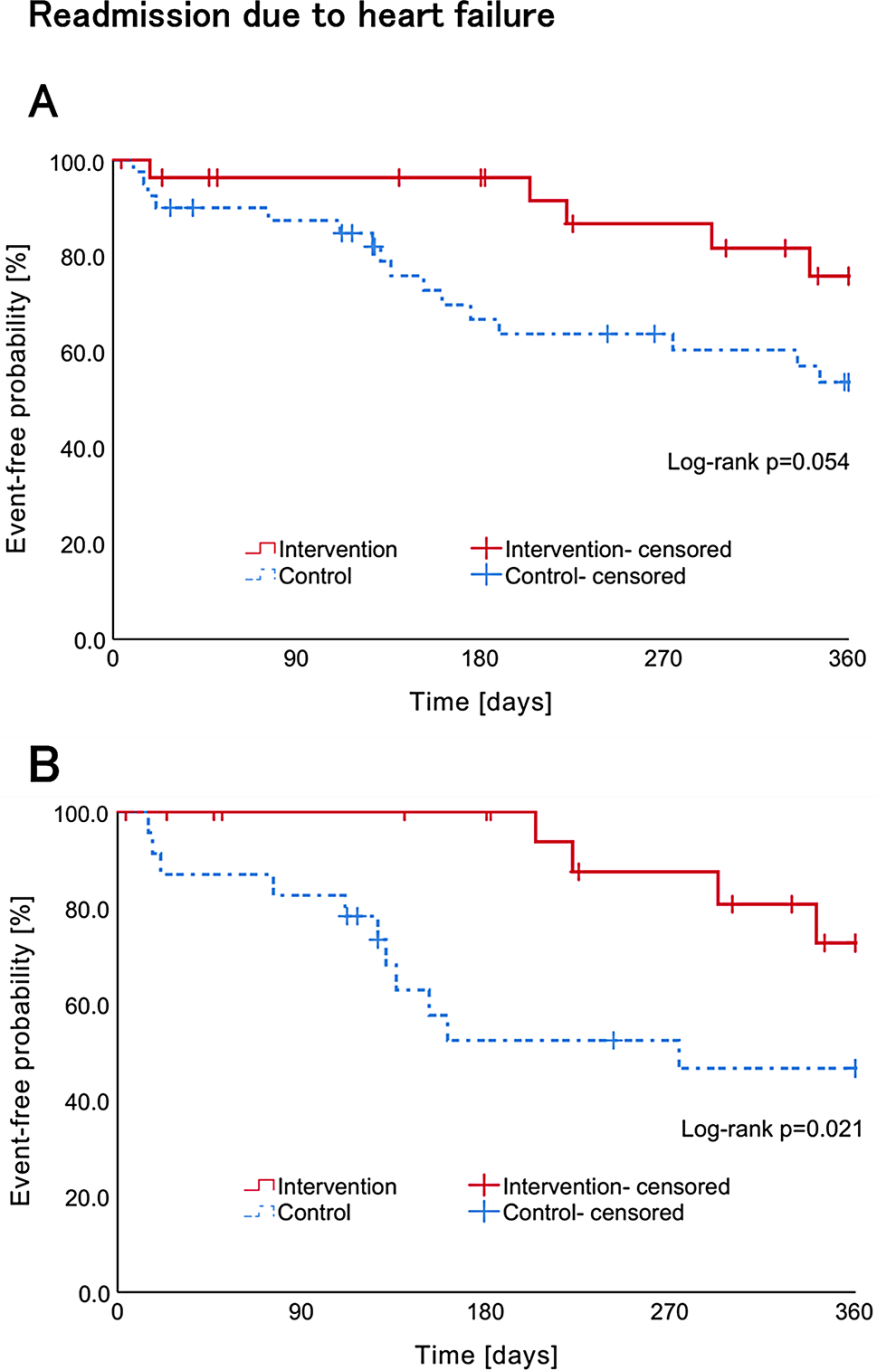
Kaplan–Meier curves for the probability of heart failure readmission in the control (**A**) and pharmacist intervention (**B**) groups before and after adjustment

The Kaplan–Meier curves for heart failure readmission at 360 days after discharge before and after adjustment are presented in Fig. 3. Prior to adjustment, during the follow-up periods (mean ± SD, 290 ± 14 [median, 297]), there were three cases (10.7%) of heart failure readmission at 270 days after discharge in the intervention group, and 13 cases (32.5%) in the control group. Additionally, there were five cases (17.9%) of heart failure readmission at 360 days after discharge in the intervention group, and 16 cases (40.0%) in the control group. The Kaplan–Meier analysis revealed that the time to readmission for heart failure 270 days after discharge was significantly longer in the intervention group than in the control group (log-rank test, *p* = 0.028). The time to readmission for heart failure at 360 days after discharge tended to be longer in the intervention group than in the control group (log-rank test, *p =* 0.054). Similarly, the results of the Cox proportional hazards model indicated that the risk of readmission for heart failure at 270 and 360 days after discharge was lower in the intervention group than in the control group, although the difference was not statistically significant (Table 3). The HR for the intervention group was 0.29 (95% CI: 0.08–1.01, *p* = 0.052) at 270 days and 0.39 (95% CI: 0.14–1.05, *p* = 0.064) at 360 days.

**Table 3:** Cox proportional hazard models for readmission due to heart failure, all-cause mortality, and the composite endpoint (hazard ratio for the control group compared to the intervention group)

	Unadjusted hazard ratio (95% CI)	Fully adjusted hazard ratio (95% CI)
HF Readmission		
During 270 days	0.29 (0.08–1.01); *p* = 0.052	0.16 (0.04–0.73); *p* = 0.018
During 360 days	0.39 (0.14–1.05); *p* = 0.064	0.28 (0.09–0.89); *p* = 0.031
All-cause mortality		
During 360 days	0.31 (0.05–2.63); *p* = 0.28	
Composite end-point		
During 270 days	0.26 (0.08–0.90); *p* = 0.033	0.17 (0.04–0.78); *p* = 0.023
During 360 days	0.37 (0.14–0.98); *p* = 0.046	0.31 (0.10–0.98); *p* = 0.047

The composite endpoint was a composite of the following events: readmission due to heart failure, all-cause mortality, hospitalization due to renal dysfunction or dehydration, and initiation of dialysis

In the model adjusted for PS, the number of heart failure readmissions at 270 days after discharge was 2 (8.7%) and 10 (43.5%) in the intervention and control groups, respectively, while the number at 360 days after discharge was 4 (17.4%) and 11 (47.8%) in the intervention and control groups, respectively. The Kaplan–Meier method revealed that the time to heart failure readmission at 270 and 360 days after discharge was significantly longer in the intervention group than in the control group (log-rank test, *p* = 0.007; log-rank test, *p* = 0.021). The COX proportional hazards model demonstrated that the risk of readmission for heart failure was significantly reduced in the intervention group relative to the control group (HR: 0.16, 95% CI: 0.04–0.73, *p* = 0.018; HR: 0.28, 95% CI: 0.09–0.89, *p* = 0.031; Table 3).

### All-cause death

Prior to adjustment, the all-cause mortality rates at 270 and 360 days post-discharge were one (3.6%) and five cases (12.5%) in the intervention and control groups, respectively. The Kaplan–Meier method revealed no statistically significant difference in the time to all-cause mortality at 360 days after discharge between the two groups (log-rank, *p* = 0.253). Similarly, the Cox proportional hazards model revealed no significant difference in the risk of all-cause mortality within 360 days of discharge (HR: 0.31, 95% CI: 0.05–2.63, *p* = 0.28; Table 3).

In the model adjusted for PS, the all-cause mortality rates at 270 and 360 days after discharge were one (4.3%) in the control group and none in the intervention group. The Kaplan–Meier method revealed no statistically significant difference between the two groups in the time to all-cause mortality at 360 days after discharge (log-rank test, *p* = 0.371). No calculation of all-cause mortality rate was performed with the Cox proportional hazard model.

### Composite endpoint

**Fig. 4:**
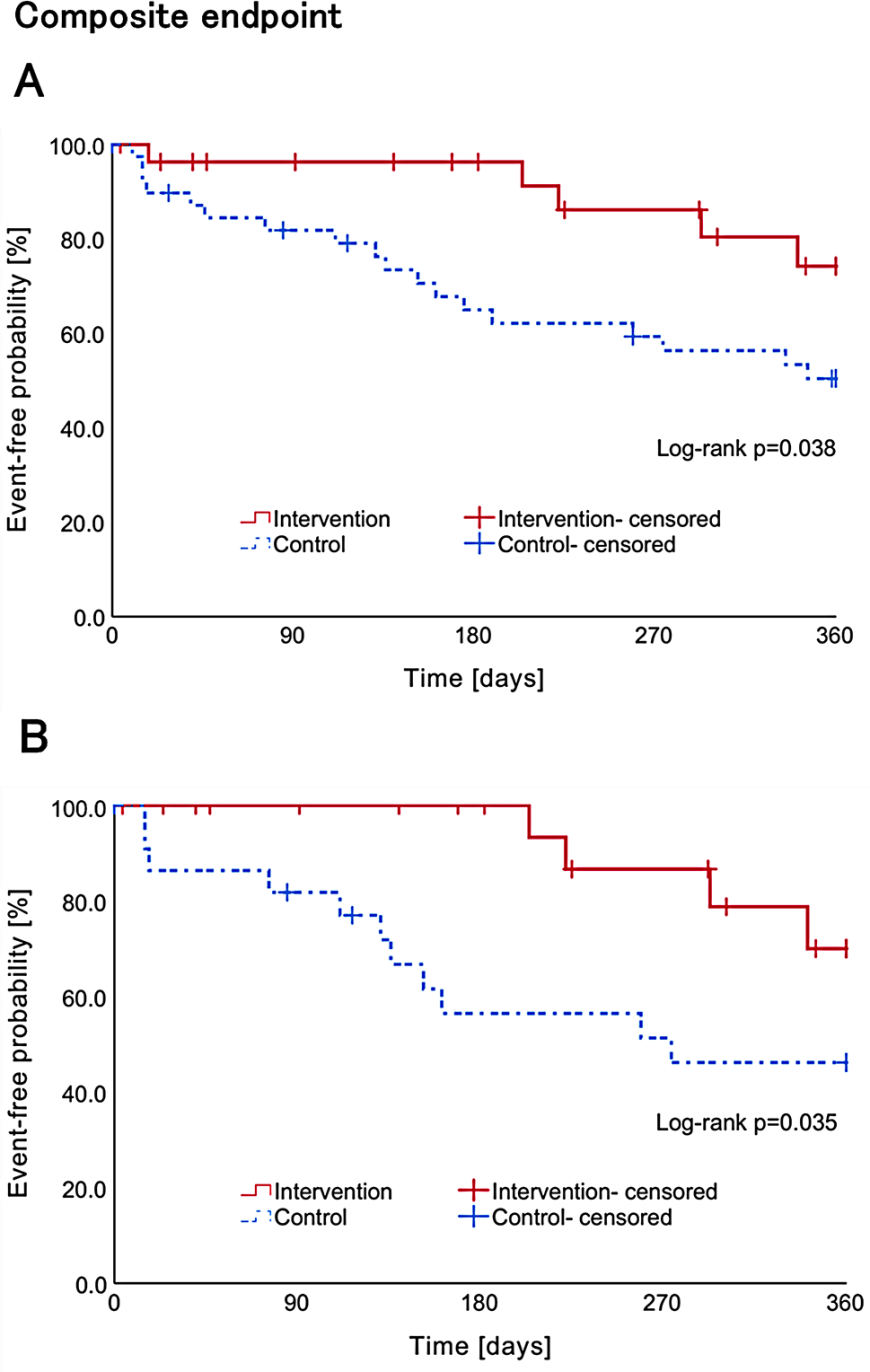
Kaplan–Meier curves for readmission due to the composite endpoint of all-cause death, first heart failure readmission, and hospitalization or dialysis due to dehydration or renal dysfunction in the control (**A**) and pharmacist intervention (**B**) group before and after adjustment

The Kaplan–Meier curves for the composite endpoint at 360 days after discharge before and after adjustment are presented in Fig. 4. Prior to adjustment, the composite endpoint at 270 days after discharge was observed in three cases (10.7%) in the intervention group and in 15 cases (37.5%) in the control group. At 360 days post-discharge, the composite endpoint was observed in five patients (17.9%) in the intervention group and 18 patients (45.0%) in the control group. The Kaplan–Meier analysis revealed that the time to the occurrence of the composite endpoint at 270 and 360 days after discharge was significantly longer in the intervention group than in the control group (log-rank test, *p* = 0.022; log-rank test, *p* = 0.038). The COX proportional hazards model demonstrated that the risk of developing the composite endpoint at 270 and 360 days after discharge was significantly reduced in the intervention group compared to the control group (HR: 0.26, 95% CI: 0.08–0.90, *p* = 0.033; HR: 0.37, 95% CI: 0.14–0.98, *p* = 0.046; Table 3).

After adjustment for PS, the composite endpoint at 270 days after discharge was observed in 2 (8.7%) and 10 cases (43.5%) in the intervention and control groups, respectively, while the composite endpoint at 360 days after discharge was observed in 4 (17.4%) and 11 cases (47.8%) in the intervention and control groups, respectively. The Kaplan–Meier analysis revealed that the time to the occurrence of the composite endpoint at 270 and 360 days after discharge was significantly longer in the intervention group than in the control group, with a significantly prolonged median time to the event (log-rank test, *p* = 0.008; log-rank test, *p* = 0.035). The COX proportional hazards model demonstrated that the risk of developing the combined endpoint at 270 days and 360 days after discharge was significantly reduced in the intervention group compared to the control group (HR: 0.17, 95% CI: 0.04–0.78, *p* = 0.023; HR: 0.31, 95% CI: 0.10–0.98, *p* = 0.047; Table 3).

## Discussion

This novel protocol for heart failure managements involving community and hospital pharmacists proved to be an effective strategy for reducing the risk of heart failure readmission, heart and kidney-related risks, and all-cause mortality. In recent years, multidisciplinary management of heart failure has been reported to reduce the occurrence of patient readmission [1–6]. In Japan, pharmacists play an important role in the management of heart failure, and they have been reported to improve the quality of life and medication adherence of patients with heart failure; however, there is little evidence that pharmacist intervention prevents heart failure [17–20]. This study is the first in Japan to show that pharmacist interventions improve hard endpoints in heart failure management.

In addition to providing regular symptom follow-ups and self-monitoring guidance by community pharmacists, this study developed a protocol for the procedure by which community pharmacists manage the addition of diuretics to the treatment regimen of patients with heart failure. Generally, acquiring prior knowledge is necessary to follow clinical protocols and make appropriate decisions for patient management; however, the introduction of protocols approved by doctors may have made it easier for community pharmacists to intervene. The clarification of follow-up items, necessary responses, and judgment criteria is beneficial for pharmacists. It is thought that establishing a reporting obligation during the follow-up period and collaborating between hospital pharmacists and medical institutions is an effective strategy for patient management.

The fact that the long-term follow-up of the intervention group was conducted by the pharmacist every 2 weeks with a high follow-up rate of 84.6% on average also suggested the usefulness of this protocol. Most cases in the intervention group yielded the desired follow-up outcomes, and communications and inquiries regarding the TRs were observed. In contrast, no special interventions beyond the provision of information regarding prescriptions were performed for the control group. In approximately half of the patients in the intervention group, additional diuretics were safely administered under the supervision of a pharmacist, which was corroborated by the finding that the composite endpoints, including readmission for heart failure, were lower in the intervention group than in the control group.

There was no significant difference between the intervention and control groups regarding the interval between outpatient visits to the cardiology department during the observation period (32 ± 18 [median, 28] days vs. 36 ± 29 [median, 29] days, t-test *p* = 0.055). This finding strongly supports the hypothesis that pharmacist follow-up and intervention, based on a protocol, positively impact the risk of heart failure prognosis.

Issues regarding the involvement of community pharmacists in heart failure management were considered during the protocol formulation process and solutions were established in advance. Community pharmacists conducted follow-up visits at least once every 2 weeks, and their role was expanded to include monitoring and improving medication adherence through strategies such as single-dose packaging and management of leftover medications.

Community pharmacist interventions have been associated with a reduced risk of readmission due to heart failure and the combined endpoints of all-cause mortality and heart and kidney diseases. Examples of patient assessments conducted by community pharmacists were observed, including proposals to reset the target body weight and respond to the situation of each patient. These observations indicate the contribution of community pharmacists to heart failure management at the community level.

In the JCARE-CARD registry study conducted in Japan, the rate of readmission for heart failure was 7% within 6 months of discharge and 35% after 1 year [21]. In the current study, the readmission rate within 360 days was 40.0% before adjustment and 47.8% after adjustment for the control group. The mean age of patients enrolled in the JCARE-CARD study was 71 years, which was lower than the mean age of patients in the present study (81 years). Furthermore, the proportion of patients with risk factors for heart failure was higher in the present study. Although there was no significant difference in baseline characteristics between the two groups prior to adjustment, numerous factors may have been risk factors for heart failure in the intervention group as a whole. The observed increase in the readmission rate in the control group after adjustment could be attributed to this factor. If this hypothesis is correct, the protocol created in this study may have suppressed the hard endpoints in patients with a relatively high risk of heart failure.

The implementation of a community-based protocol that clearly defined the methods of intervention for community pharmacists in heart failure management was demonstrated to be an effective means of reducing heart failure-related events, all-cause mortality, and cardiac and renal-related events.

### Study limitations

This study was conducted at a single center and the number of patients was relatively limited. Although patients and pharmacies were allocated in an attempt to ensure even distribution, this was an open-label study that did not employ randomization for allocation; hence, PS matching was used to minimize discrepancies in baseline characteristics between the two groups. While it is believed that matching was conducted effectively through balance checks, the balance and impact of the unmeasured variables could not be verified. Furthermore, concerning readmission due to heart failure, the focus of this study, the attending physician determined the patient’s hospitalization based on daily indicators. It is important to consider that this study was open-label and did not clearly define the criteria for hospitalization.　To obtain more reliable results, studies with larger sample sizes at multiple sites should be conducted.

## Conclusions

In this study, we developed a novel approach for chronic heart failure management through a collaborative effort between hospitals and community　pharmacists. Adherence to this protocol may considerably reduce the likelihood of heart failure development in patients with existing cardiovascular conditions.

## Data Availability

No datasets were generated or analysed during the current study.

## Abbreviations

BMI: Body mass index
CABG: Coronary artery bypass grafting
CI: Confidence intervals
HR: Hazard ratio
LVEF: Left ventricular ejection fraction
NT-proBNP: N-terminal pro-B-type natriuretic peptide
PCI: Percutaneous coronary intervention
PPS: Per protocol set
PS: Propensity scores
TR: Tracing report
