# Correction: The impact of a heart failure management protocol based on a hospital-community pharmacist collaboration

**DOI:** 10.1186/s40780-025-00433-6

**Published:** 2025-04-01

**Authors:** Junichi Terashima, Takahiro Kambara, Eisei Hori, Masahiro Fukatsu, Yukina Ichiki, Eri Oki, Risako Koketsu, Rika Taguchi, Suzuka Mii, Ryoka Hiro, Teruhiro Sakaguchi, Hiroyuki Osanai, Tomoya Tachi, Tadashi Suzuki

**Affiliations:** 1 Department of Pharmacy, Tosei General Hospital, 160, Nishioiwake-cho, Seto, 489-8642 Aichi Japan; 2 Department of Clinical Pharmacy, Graduate School of Pharmaceutical Sciences, Nagoya City University, 3-1, Tanabedori, Mizuho-ku, Nagoya, 467-8603 Aichi Japan; 3 Department of Cardiovascular Medicine, Tosei General Hospital, 160, Nishioiwake-cho, Seto, 489-8642 Aichi Japan; 4 Education and Research Center for Clinical Pharmacy, Faculty of Pharmaceutical Sciences, Nagoya City University, 3-1, Tanabedori, Mizuho-ku, Nagoya, 467-8603 Aichi Japan

**Journal of Pharmaceutical Health Care and Sciences (2025) 11:19**

10.1186/s40780-025-00426-5

Affiliations 2 and 3 were incorrectly swapped in the original publication of this article. The incorrect and correct information is listed in this correction article. The original article has been updated.

Incorrect

^2^Department of Cardiovascular Medicine, Tosei General Hospital, 160, Nishioiwake-cho, Seto, Aichi 489–8642, Japan

^3^Department of Clinical Pharmacy, Graduate School of Pharmaceutical Sciences, Nagoya City University, 3 − 1, Tanabedori, Mizuho-ku, Nagoya, Aichi 467–8603, Japan

Correct

^2^Department of Clinical Pharmacy, Graduate School of Pharmaceutical Sciences, Nagoya City University, 3 − 1, Tanabedori, Mizuho-ku, Nagoya, Aichi 467–8603, Japan

^3^Department of Cardiovascular Medicine, Tosei General Hospital, 160, Nishioiwake-cho, Seto, Aichi 489–8642, Japan

